# Activation of neutral-sphingomyelinase, MAPKs, and p75 NTR-mediating caffeic acid phenethyl ester–induced apoptosis in C6 glioma cells

**DOI:** 10.1186/1423-0127-21-61

**Published:** 2014-07-05

**Authors:** Tsui-Hwa Tseng, Chien-Heng Shen, Wen-Shih Huang, Cheng-Nan Chen, Wen-Hai Liang, Tseng-Hsi Lin, Hsing-Chun Kuo

**Affiliations:** 1 School of Medical Applied Chemistry, Chung Shan Medical University, No. 110, Section 1, Chien-Kuo N. Road, Taichung 402, Taiwan; 2 Department of Medical Education, Chung Shan Medical University Hospital, No. 110, Section 1, Chien-Kuo N. Road, Taichung 402, Taiwan; 3 Department of Hepato-Gastroenterology, Chang Gung Memorial Hospital, Chiayi, Taiwan; 4 Graduate Institute of Clinical Medical Sciences, College of Medicine, Chang Gung University, Taoyuan, Taiwan; 5 Division of Colon and Rectal Surgery, Department of Surgery, Chang Gung Memorial Hospital, Chiayi, Taiwan; 6 Department of Biochemical Science and Technology, National Chiayi University, Chiayi, Taiwan; 7 Institute of Biochemistry and Biotechnology, Chung Shan Medical University, Taichung, Taiwan; 8 Division of Transfusion Medicine, Department of Pathology and Laboratory Medicine; Division of Hematology, Department of Internal Medicine, Taichung Veterans General Hospital, Taichung, Taiwan; 9 Department of Internal Medicine, School of Medicine, Chung Shan Medical University, Taichung, Taiwan; 10 Institute of Nursing and Department of Nursing, Chang Gung University of Science and Technology, Chiayi, Taiwan; 11 Chronic Diseases and Health Promotion Research Center, Chang Gung University of Science and Technology, Chiayi, Taiwan; 12 Research Center for Industry of Human Ecology, Chang Gung University of Science and Technology, Taoyuan, Taiwan

**Keywords:** Caffeic acid phenethyl ester, C6 glioma cells, Neutral sphingomyelinase, p75 neurotrophin receptor, MAPK, Nerve growth factor

## Abstract

**Background:**

Caffeic acid phenethyl ester (CAPE), a component of propolis, is reported to possess anti-inflammatory, anti-bacterial, anti-viral, and anti-tumor activities. Previously, our laboratory demonstrated the *in vitro* and *in vivo* bioactivity of CAPE and addressed the role of p53 and the p38 mitogen-activated protein kinase (MAPK) pathway in regulating CAPE-induced apoptosis in C6 glioma cells.

**Results:**

C6 cancer cell lines were exposed to doses of CAPE; DNA fragmentation and MAPKs and NGF/P75NTR levels were then determined. SMase activity and ceramide content measurement as well as western blotting analyses were performed to clarify molecular changes. The present study showed that CAPE activated neutral sphingomyelinase (N-SMase), which led to the ceramide-mediated activation of MAPKs, including extracellular signal-regulated kinase (ERK), Jun N-terminus kinase (JNK), and p38 MAPK. In addition, CAPE increased the expression of nerve growth factor (NGF) and p75 neurotrophin receptor (p75NTR). The addition of an N-SMase inhibitor, GW4869, established that NGF/p75NTR was the downstream target of N-SMase/ceramide. Pretreatment with MAPK inhibitors demonstrated that MEK/ERK and JNK acted upstream and downstream, respectively, of NGF/p75NTR. Additionally, CAPE-induced caspase 3 activation and poly [ADP-ribose] polymerase cleavage were reduced by pretreatment with MAPK inhibitors, a p75NTR peptide antagonist, or GW4869.

**Conclusions:**

Taken together, N-SMase activation played a pivotal role in CAPE-induced apoptosis by activation of the p38 MAPK pathway and NGF/p75NTR may explain a new role of CAPE induced apoptosis in C6 glioma.

## Background

Caffeic acid phenethyl ester (CAPE) is secondary products of propolis and thus possesses anti-oxidant, anti-angiogenic, anti-viral, anti-inflammatory, and anti-metastatic properties. CAPE also selectively represses the proliferation of several types of carcinoma cells, but show almost no toxic effects on normal peripheral blood cells or on normal hepatocytes [1-6]. Studies of previous studies have revealed a broad spectrum of biological activities that CAPE induced apoptosis in oral cancer cells and glioma cells [7,8]. In *in vitro* and *in vivo* studies, CAPE inhibited the proliferation of C6 glioma cells [9]. Further, CAPE enhanced all-trans retinoic acid-induced differentiation in human leukemia HL-60 cells [10]. The mitogen-activated protein kinases (MAPKs) are a family of protein kinases that comprise a diverse superfamily of phylogenetically conserved serine/threonine kinases. There are three classical MAP kinase families: c-Jun N-terminal kinases (JNKs), Ras/extracellular signal-regulated kinase (ERK), and p38 MAPK. Although it is previously showed that activation of ERK1/2 leads to cell growth, ERK1/2 activation results in cell apoptosis under some conditions [11,12]. JNK1/2 and p38 MAPK are highly effected in signalling to various stress signals, including TNFα, oxidative stress, and ultraviolet (UV) light. Their activation is most frequently associated with the induction of apoptosis [13,14]. Our previous study showed that CAPE caused p53-dependent apoptosis in C6 glioma cells through the p38 MAPK signaling pathway [8]. In addition to activating p38 MAPK in C6 glioma cells, CAPE increased the phosphorylation of ERK and JNK, whose involvement was previously unknown.

Nerve growth factor (NGF) regulates neurotrophic actions on many neurons in rats [15]. NGF are involved a surprising variety of neurons, glia, and nonneural cells by a high-affinity receptor TrkA and a low-affinity receptor, p75 neurotrophin receptor (p75NTR) [16]. TrkA and p75NTR collaborate to essentially takes place upon the binding to the cell surface as neurotrophins [17]. It is now thought that p75NTR play a crucial role in the glioma apoptotic pathway [18]. p75NTR cognate TNFα superfamily receptors Fas and CD40 are expressed in tissues to which these glioma cells commonly death [19].

Three mammalian isoforms of neutral sphingomyelinase (N-SMase) have been cloned to date. N-SMase is membrane-bound and Mg^2+^-dependent. Acidic sphingomyelinase (A-SMase) has three isoforms, an endosomal lysosomal A-SMase, a secretory Zn^2+^-dependent A-SMase, and a receptor-activated A-SMase [20]. A ceramide is composed of sphingosine and a fatty acid that serves as a proapoptotic molecule [21]. Ceramide has been involved in a variety of physiological functions including apoptosis, cell growth arrest, differentiation, cell migration and adhesion. Several studies have attempted to define the roles of SMase and ceramide on induction of NGF synthesis in primary astrocyte cultures, indicating it may be crosstalk between ceramide and NGF receptor (NGFR) signaling in the nervous cells [22]. Further, N-SMase plays a role in chemotherapy-mediated cell death. In the present study, we examined whether SMase/ceramide induced up-regulation of NGF/p75NTR is mediated by CAPE-induced apoptosis, and we clarified the relationship between SMase/ceramide, NGF/p75NTR, and the MAPK signaling pathway in C6 glioma cells.

## Methods

### Chemical reagents and antibodies

All culture materials were purchased from Invitrogen (Carlsbad, CA). The Amplex Red Sphingomyelinase kit was purchased from Sigma (St. Louis, MO, USA). Sodium dodecyl sulfide (SDS), bis-acrylamide, ammonium persulfate, N,N,N’,N’-tetramethylethylenediamine (TEMED), and nitrocellulose (NC) paper were from Bio-Rad (Hercules, CA). Caffeic acid phenethyl ester, Triton X-100, Tris base, β-actin antibody, non-hydroxy fatty acid ceramide, and 4’,6-diamidino-2-phenylindole (DAPI) were from Sigma (St. Louis, MO). GW4869, a specific inhibitor of N-SMase, was also purchased from Sigma. Antibodies against NGF, p75NTR, Trk, poly [ADP-ribose] polymerase (PARP), and caspase-3 were from Santa Cruz Biotechnology (Santa Cruz, CA). The p75 antagonist peptide YCDIKGKECY (the cysteine-cysteine bond that results in cyclization is underlined), which is reportedly specific to p75NTR, was synthesized by Protech Technology (Reno, NV) to a minimum of 90% purity [23]. High-performance thin-layer chromatography (HPTLC) silica gel 60 plates were obtained from Merck (Whitehouse Station, NJ).

### Cell culture

The rat C6 glioma cell line was originally derived from an *N*-nitrosomethylurea-induced rat brain tumor [24]. Cells were cultured in minimal essential medium supplemented with 10% fetal calf serum and antibiotics (100 U/ml of penicillin and 100 mg/ml of streptomycin) at 37°C in a humidified atmosphere of 5% CO_2_ and 95% air. All experiments were performed in plastic tissue culture flasks and dishes or in microplates (Nunc, Naperville, Denmark).

### Assessment of cell viability

Cell viability was assessed with the MTT colorimetric assay. The cells were seeded at 2 × 10^4^ cells/ml and incubated with various concentrations of CAPE (0, 5, 10, 25, 50, and 100 μM) for 24 and 48 h. The medium was changed, and the cells were incubated with 3-(4,5-dimethylthiazol-2-yl)-2,5-diphenyltetrazolium bromide (MTT; 0.5 mg/ml) for 4 hr. The viable cell number was directly proportional to the production of formazan, which was solubilized with isopropanol and measured spectrophotometrically at 563 nm.

### Analysis of DNA fragmentation

C6 glioma cells cultured in 100-mm dishes were resuspended in 200 ml of hypotonic lysis buffer (0.2% Triton X-100, 1 mM ethylenediaminetetraacetic acid (EDTA), 10 mM Tris–HCl, pH 7.5) and incubated for 20 min at 4°C. After centrifugation for 5 min at 14,000 × *g*, the supernatant was collected, incubated with RNase A (400 mg/ml) for 30 min at 37°C, and digested with proteinase K (200 mg/ml) for 30 min at 50°C. The fragmented DNA was then precipitated overnight at −20°C in 50% isopropanol and 0.5 M NaCl and centrifuged for 10 min at 14,000 × *g*. The dried pellets were dissolved in 20 ml of ultra-pure water, and DNA was separated by electrophoresis on a 1.8% agarose gel at 50 V for 1 h.

### Preparation of total cell extracts and immunoblot analysis

Cells were plated on 10-cm^2^ dishes at a density of 1 × 10^6^ cells/ml in the presence or absence of CAPE (50 μM) for 0–24 h and harvested. To prepare whole-cell extracts, cells were washed with phosphate-buffered saline (PBS) plus zinc ion (1 mM) and suspended in lysis buffer (50 mM Tris, 5 mM EDTA, 150 mM NaCl, 1% NP-40, 0.5% deoxycholic acid, 1 mM sodium orthovanadate, 81 μg/ml aprotinin, 170 mg/ml leupeptin, 100 mg/ml phenylmethylsulfonyl fluoride (PMSF); pH 7.5). Lysates were mixed for 30 min at 4°C and centrifuged at 10,000 × *g* for 10 min. The supernatants were collected as whole-cell extracts. The protein content was determined with Bio-Rad protein assay reagent using bovine serum albumin as a standard. Western blotting with enhanced chemiluminescence (ECL) detection was performed as follows. Total cell lysates with equal protein content from control and CAPE-treated samples was resolved on 8%–12% SDS-PAGE gels along with a prestained protein molecular weight standard (Bio-Rad). Proteins were then blotted onto NC membranes (Sartorious) and reacted with primary antibodies (anti-caspase-3, anti-PARP, anti-Trk, anti-NGF, anti-p75, anti-ERKs, anti-JNKs, anti-p38 MAPK, anti-phosphoserine, anti-phospho-ERKs (Thr183/Tyr185), anti-phospho-JNKs (Thr183/Tyr185), and anti-phospho-p38 (Thr180/Tyr182), and anti-phospho-Trk from Santa Cruz Biotechnology (Santa Cruz, CA); anti-β-actin from Sigma as an internal control). The secondary antibody was a peroxidase-conjugated goat anti-mouse antibody. After antibody binding, the bands were visualized using a commercial ECL kit.

### SMase activity assay

SMase activity was analyzed with an Amplex Red Sphingomyelinase Assay Kit. Cells were washed with PBS and pelleted by centrifugation at 1500 × *g* for 10 min at 4°C. The cell pellet was resuspended, lysed in buffer containing 1% Triton X-100, 1 μg/ml aprotinin, 1 mM EDTA, and 100 μg/ml PMSF for 60 min on ice, and then centrifuged at 17,000 × *g* for 10 min at 4°C to remove the nuclei. The protein concentration in the supernatant was measured with the Bio-Rad Protein Assay. To analyze the activity of N-SMase, the supernatant was diluted with 1× reaction buffer containing 0.1 M Tris–HCl and 10 mM MgCl_2_, pH 7.4, and then inoculated into 96-well plates (100 μl/well). The total amount of protein in each well was 25 μg. The solution was added to 100 μl of working solution (containing 8 U/ml alkaline phosphatase, 0.2 U/ml choline oxidase, 2 U/ml horseradish peroxidase, and 0.5 mM sphingomyelin) and incubated for 45 min at 37°C. The H_2_O_2_ generated reacted with Amplex Red to elicit fluorescent resorufin. The fluorescent intensity was measured with a fluorescence multi-well reader (HTS 7000) immediately at excitation/emission wavelengths of 571/585 nm. Acid sphingomyelinase (A-SMase) activity was analyzed with a two-step assay. First, the supernatant was diluted with 50 mM sodium acetate (not 1× reaction buffer), and 100 μl of the diluted supernatant was inoculated to each well of a 96-well plate. To generate phosphorylcholine and ceramide, 10 μl of 5 mM sphingomyelin solution was added to each well and then incubated for 1 h at 37°C. Working solution (100 μl) containing 8 U/ml alkaline phosphatase, 0.2 U/ml choline oxidase, and 2 U/ml horseradish peroxidase was then added to each well and reacted with phosphorylcholine for 45 min at 37°C. The H_2_O_2_ generated from the two-step procedure then reacted with Amplex Red to elicit resorufin, which was measured with a fluorescence multi-well reader as described above.

### Quantitative measurement of ceramide content

Cells were harvested in lysis buffer containing 50 mM Tris–HCl, pH 6.8, 10% glycerol, 2% SDS, and 5% β-mercaptoethanol and vortexed for 1 hr at 4°C. The lipids were extracted by the following procedure. In brief, 1 ml of cell lysate was suspended with 1.25 ml of chloroform and 2.5 ml of methanol, vortexed, and incubated overnight at 4°C. After centrifugation for 5 min at 1700 × *g*, 1.25 ml of chloroform and 1.25 ml of 0.88% KCl were added to the supernatant. The tubes were then vortexed and centrifuged for 5 min at 1700 × *g*. The organic lower phase was transferred to a fresh tube and evaporated. The lipids were dissolved into chloroform-methanol [2:1 (v/v)] and analyzed using an HPTLC silica gel 60 plate. Dichloromethane-methanol-glacial acetic acid [100:2:5 (v/v)] was used to separate the lipid. Individual lipid classes were visualized by dipping each plate into CuSO_4_ (3%)/H_3_PO_4_ (8%) for 30 sec. The plate was then incubated in an oven at 180°C for 10 min. The bands were scanned and the data were evaluated with an LAS-3000 imager (Fuji Photo Film Co., Ltd., Tokyo, Japan). In addition to the three sample replicates, C_2_-ceramide was added to each plate as the lipid standard.

### Determining the effects of MAPK inhibitors, SMase inhibitors, and a p75NTR antibody inhibitor

C6 glioma cells were pretreated with 50 μM PD98059, 5 μM SB203580, 10 μM SP600125, 10 or 20 μM GW4869, or a p75NTR antibody inhibitor for 1 hr and then treated with 50 μM CAPE for 0–24 hr. The cell extracts were analyzed by immunoblotting with specific antibodies (anti-CPP32, anti-PARP, anti-NGF, anti-p75, anti-ERKs, anti-JNKs, anti-p38 MAPK, anti-phosphoserine, anti-phospho-ERKs (Thr183/Tyr185), anti-phospho-JNKs (Thr183/Tyr185), and anti-phospho-p38 (Thr180/Tyr182), as previously described.

### DAPI staining

We used DAPI staining to assess morphological changes in chromatin structure in C6 glioma cells undergoing apoptosis. Cells were trypsinized, mounted on glass slides, fixed in 4% paraformaldehyde for 30 min, and stained with 1 μg/ml DAPI for 30 min. Apoptosis was characterized by chromatin condensation and fragmentation, which were visualized with fluorescence microscopy.

### Statistical analysis

Data were reported as the mean ± SD of three independent experiments and evaluated by one-way ANOVA. Differences were considered significant at *P* < 0.05.

## Results

### Involvement of MAPKs in CAPE-mediated caspase 3 activation and PARP cleavage

**Figure 1:**
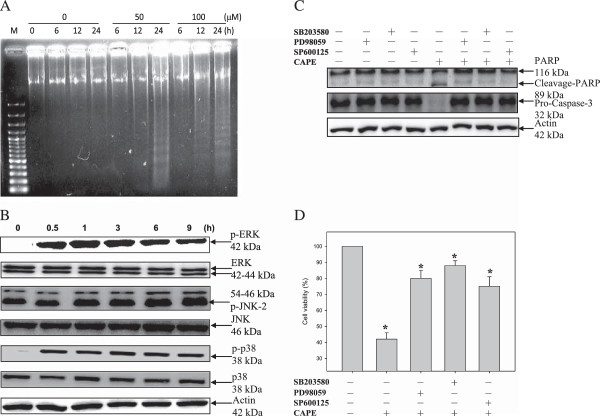
**Involvement of MAPK activation in CAPE-induced apoptosis. (A)** CAPE induced DNA fragmentation in C6 glioma cells. Cells were treated with or without CAPE (50 and 100 μM) for the indicated times. DNA was separated on a 1.8% agarose gel, stained with ethidium bromide, and photographed under ultraviolet light. **(B)** Effect of CAPE on the activation of MAPKs. Cells were cultured in medium containing CAPE (50 μM) for the indicated times. Following treatment, cells were harvested, and cell lysates were prepared for western blot analysis using anti-phospho-ERK, anti-phospho-JNK, anti-phospho-p38, anti-ERK, anti-JNK, anti-p38, and anti-actin antibodies. **(C)** Effect of MAPK inhibitors on CAPE-induced caspase 3 activation and PARP cleavage. C6 glioma cells were pretreated with MAPK inhibitors for 1 h and then treated with CAPE for 24 h. Cell extracts were assessed by western blot analysis using anti-PARP and anti-procaspase 3 antibodies. The bands were visualized with an ECL reagent and quantified by densitometry. The data is representative of two independent experiments. **(D)** Effect of MAPK inhibitors on CAPE-induced C6 cells viability. The data are shown as the percentage of the control group. Data are presented as the mean ± S.D. of three independent experiments. *P < 0.05, compared with control.

Consistent with the results of our previous paper, treatment with CAPE (50 μM) for 24 h induced apoptosis [8] (Figure 1A). In addition, immunoblot analysis with antibodies against the phosphorylated forms of three kinases showed that CAPE treatment activated ERK, JNK, and p38 MAPK (Figure 1B). ERK and p38 MAPK were activated after treatment with CAPE (50 μM) for 0.5 h, and JNK was activated after treatment for 6 h. The MAPK signaling inhibitors PD98059, SB203580, and SP600125 blocked CAPE-mediated caspase 3 activation and PARP cleavage (Figure 1C). Caspase 3 plays an essential role in executing apoptosis. PARP, an enzyme involved in DNA repair, genome surveillance, and integrity, is a substrate of caspase 3. As shown in Figure 1, The MAPK signaling inhibitors, significantly reversed the CAPE-inhibited viability at 24 h. These results implied that all three MAPKs were involved in CAPE-induced apoptotic death in C6 glioma cells.

### Involvement of NGF/p75NTR in CAPE-mediated caspase 3 activation and PARP cleavage

**Figure 2:**
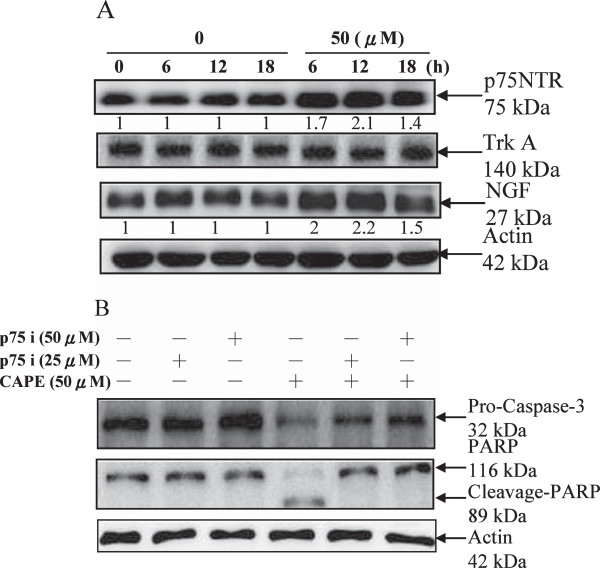
**Effect of CAPE-induced NGF/p75NTR activation on apoptosis in C6 glioma cells. (A)** Effect of CAPE on the expression of p75NTR, TrkA, and NGF in C6 glioma cells. Cells were treated with CAPE (50 μM) for the indicated times. Protein in the total cell lysate was detected with anti-p75NTR, anti-TrkA, anti-NGF, and anti-actin antibodies. Bands were visualized with an ECL reagent and quantified. **(B)** Inhibition of CAPE-induced caspase-3 and PARP expression by the anti-p75NTR antagonist peptide. C6 glioma cells were pretreated with the anti-p75NTR inhibitor (25 and 50 μM) for 1 h and then treated with CAPE (50 μM) for 24 h. Cell extracts were assessed by western blot analysis with anti-procaspase-3, anti-PARP, and anti-actin antibodies. Bands were visualized with an ECL reagent and quantified by densitometry. The data is representative of two independent experiments.

C6 cells were treated with or without 50 μM CAPE for different lengths of time. NGF increased 2.2-fold, and p75NTR increased 2.1-fold when compared with protein levels in control cells. TrkA, another NGF receptor, was unchanged at 12 h (Figure 2A). To examine whether the NGF/p75NTR interaction was involved in CAPE-induced apoptotic death, cells were treated with 50 μM CAPE in the presence or absence of the p75NTR antagonist peptide (p75 i) for 24 h. The p75NTR antagonist peptide reduced CAPE-mediated caspase 3 activation and PARP cleavage (Figure 2B).

### Association of MAPKs and NGF/p75NTR after treatment with CAPE

**Figure 3:**
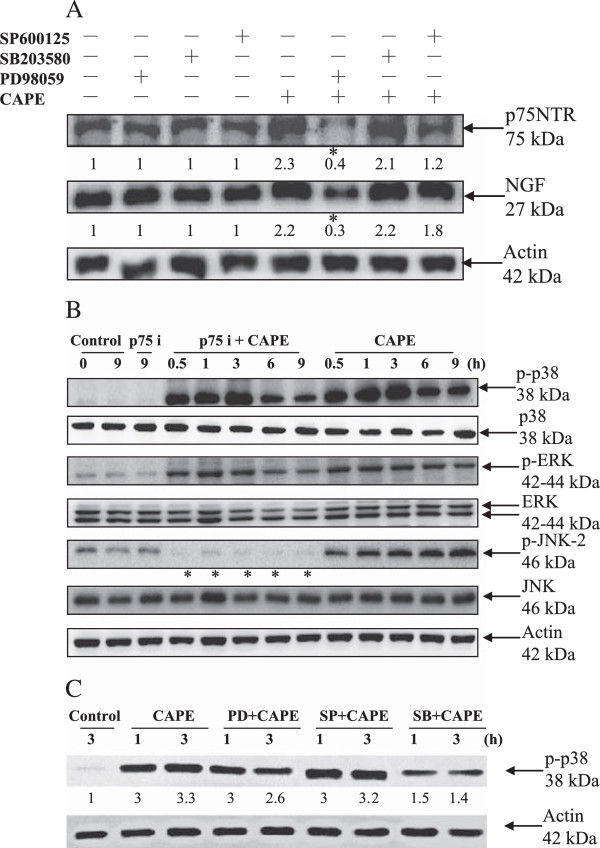
**The relationship between MAPKs and NGF/p75NTR after treatment with CAPE in C6 glioma cells. (A)** Effect of MAPK inhibitors on CAPE-induced NGF and p75NTR expression. C6 glioma cells were pretreated with a JNK inhibitor (SP600125, 10 μM), p38 inhibitor (SB20358, 10 μM), or MEK/ERK inhibitor (PD98059, 10 μM) for 1 h and then treated with CAPE (50 μM) for 12 h. Cell extracts were assessed by western blot analysis using anti-NGF, anti-p75NTR, and anti-actin antibodies. **(B)** Effect of the anti-p75NTR antagonist on CAPE-induced MAPK activation. C6 glioma cells were pretreated with the anti-p75NTR antagonist peptide for 1 h and then treated with CAPE for the indicated times. Cells extracts were assessed by western blot analysis using anti-phospho-p38, anti-phospho-ERK, anti-phospho-JNK, anti-p38, anti-ERK, anti-JNK, and anti-actin antibodies. Bands were visualized with ECL reagent and quantified by densitometry. The data is representative of two independent experiments. *, apparent reduction in expression. **(C)** Effect of MAPK inhibitors on CAPE-inducedp38 activation. Whole cell lysates from CAPE-treated C6 cells by western blotting analysis using antibodies against the phosphorylated forms of p38 (Thr180/Tyr182) and anti-actin.

Figure 3A shows C6 cells treated with 50 μM CAPE in the presence or absence of MAPK inhibitors for 12 h. PD98059, an inhibitor of the MEK/ERK signaling pathway, reduced NGF and p75NTR expression, but p38 MAPK inhibitor SB203580 and JNK inhibitor SP600125 had no effect. In addition, cells were pretreated with or without the p75NTR antagonist peptide, and 50 μM CAPE was then added for the indicated times. Phosphorylation of JNK was reduced in cells treated with the p75NTR antagonist peptide (Figure 3B). The results indicated that ERK and JNK acted upstream and downstream, respectively, of NGF/p75NTR. To determine the effect of PD98059 (PD) or SP600125 (SB) also block CAPE induced p38 activation, we assessed whole cell lysates from CAPE-treated C6 cells by western blotting analysis. SB203580 (SB) showed significantly inhibited the CAPE-induced phosphorylation of p38 but PD98059 (PD) and SP600125 (SP) were not (Figure 3C).

### Activation of N-SMase by CAPE

**Figure 4:**
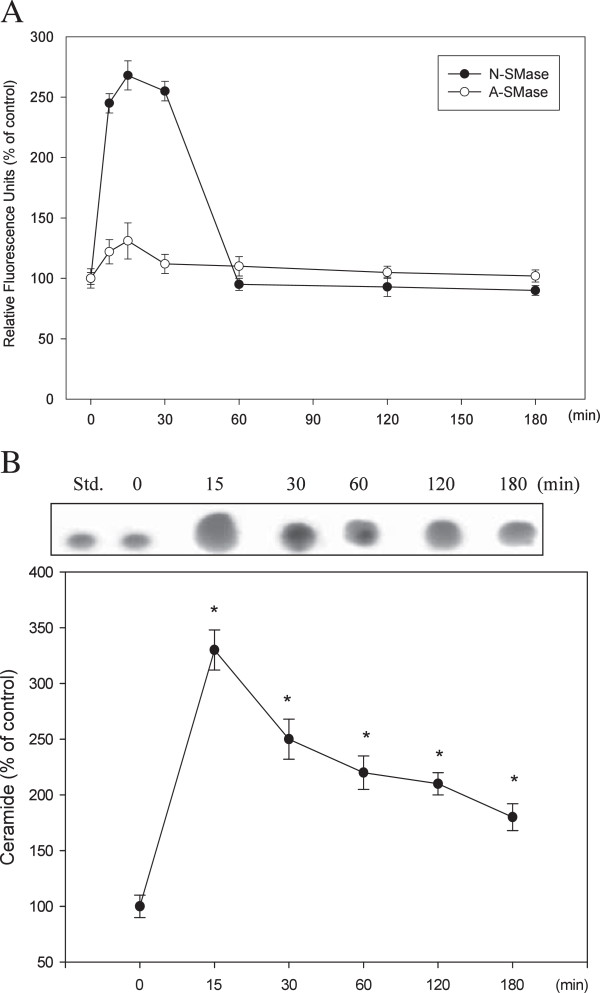
**Effect of CAPE on the SMase/ceramide pathway. (A)** Time course showing the effect of CAPE on intracellular SMase activation in C6 glioma cells. Cells were treated with CAPE (50 μM) for the indicated times, and SMase activation was analyzed using a fluorometric assay in which SMase activity was directly proportional to the fluorescence emitted. Data are presented as the mean ± S.D. of three independent experiments. **(B)** Time course showing the effect of CAPE on intracellular ceramide levels in C6 glioma cells. Cells were treated with CAPE (50 μM) for the indicated times, and ceramide was resolved by HPTLC. Std, standard (C-2 ceramide). The ceramide levels are shown as the percentage of the control group at 0 min. Data are presented as the mean ± S.D. of three independent experiments. *P < 0.05, compared with 0 min.

C6 glioma cells were treated with 50 μM CAPE for 0–180 min. SMase activity was calculated as the percentage of fluorescence. Figure 4A shows that N-SMase activity peaked at 15 min and then declined within 60 min, whereas A-SMase was only slightly activated by CAPE. Figure 4B shows the change in ceramide levels. After CAPE treatment, ceramide increased roughly 3.3-fold within 15 min and 2-fold within 180 min.

### MAPKs and NGF/p75NTR signal downstream of N-SMase

**Figure 5:**
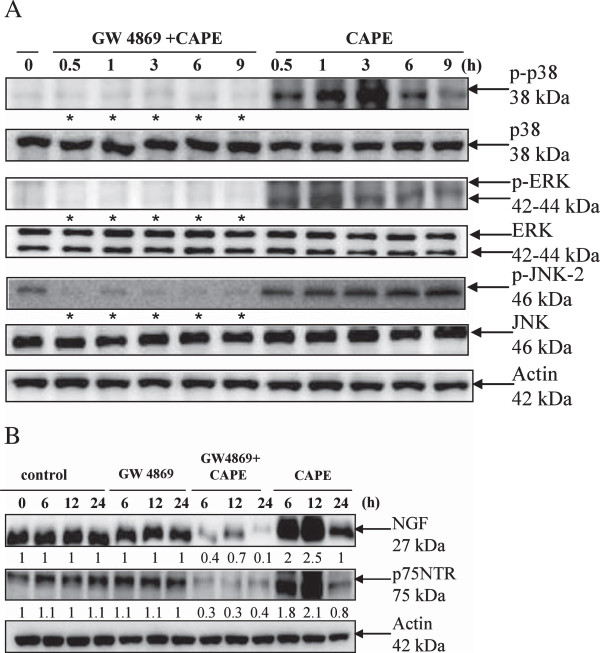
**Effect of N-SMase on CAPE-induced MAPK activation and NGF/p75NTR expression. (A)** Inhibition of CAPE-induced phosphorylation of MAPKs by GW4869. C6 glioma cells were pretreated with a sphingomyelinase inhibitor (GW4869, 20 μM) for 1 h and then treated with CAPE (50 μM) for the indicated times. Cell extracts were assessed by western blot analysis with anti-phospho-p38, anti-p38, anti-phospho-ERK, anti-ERK, anti-phospho-JNK, anti-JNK, and anti-actin antibodies. Bands were visualized with ECL reagent and quantified by densitometry. The data is representative of two independent experiments. *, apparent reduction in expression. **(B)** Inhibition of CAPE-induced NGF and p75NTR expression by a sphingomyelinase inhibitor. C6 glioma cells were pretreated with a sphingomyelinase inhibitor (GW4869, 20 μM) for 1 h and then treated with CAPE (50 μM) for the indicated times. Cell extracts were assessed by western blot analysis with anti-NGF, anti-p75NTR, and anti-actin antibodies.

Figure 5A shows that GW4869, an N-SMase inhibitor, almost blocked the phosphorylation of MAPKs induced by CAPE. In addition, GW4869 treatment attenuated the increase in NGF and p75NTR expression induced by CAPE treatment (Figure 5B). These data identify MAPKs and NGF/p75NTR as downstream signals in CAPE-induced N-SMase activation.

### CAPE induces apoptosis through N-SMase activation

**Figure 6:**
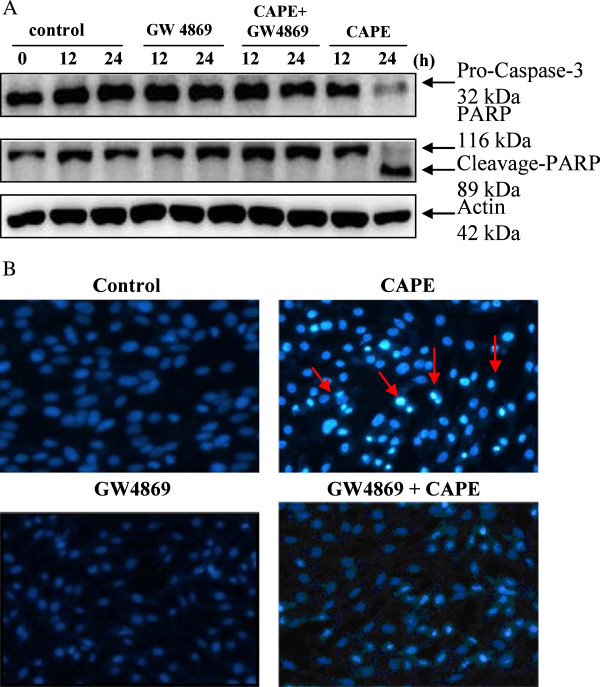
**Critical role of N-SMase in CAPE-induced apoptosis. (A)** Inhibition of CAPE-induced caspase-3 and PARP expression by a sphingomyelinase inhibitor. C6 glioma cells were pretreated with a sphingomyelinase inhibitor (GW4869, 20 μM) for 1 h and then treated with CAPE (50 μM) for the indicated times. Cell extracts were assessed by western blot analysis with anti-procaspase-3, anti-PARP, and anti-actin antibodies. **(B)** C6 glioma cells were pretreated with a sphingomyelinase inhibitor (GW4869, 20 μM) for 1 hand and then treated with CAPE (50 μM) for 24 h. Apoptosis was detected with DAPI staining.

To confirm that N-SMase activation plays a role in CAPE-induced apoptosis, cells were treated with 50 μM CAPE in the presence or absence of GW4869 for 24 h. GW4869 attenuated caspase 3 activation and PARP cleavage in CAPE-treated cells. In addition, the apoptotic death in C6 cells was analyzed by DAPI staining. Strong blue fluorescence (indicative of chromatin condensation) was visible in CAPE-treated apoptotic cells. In contrast, fluorescence was weak in control cells, cells treated with GW4869, and cells treated with GW4869 plus CAPE (Figure 6B). These results indicate that N-SMase plays a critical role in CAPE-induced apoptosis.

## Discussion

Apoptosis, an active process that leads to cell death, is mediated by programmed signaling pathways that can be initiated by various extracellular or intracellular stimuli. The ability to efficiently induce apoptosis is essential for the development of effective cancer therapies. In the present study, we demonstrated that CAPE-induced apoptosis involves the activation of N-SMase and the accumulation of ceramide. Blocking the activation of N-SMase decreased CAPE-induced caspase 3 activation and PARP cleavage. These results demonstrate the pivotal role of N-SMase in CAPE-induced apoptosis. SMases are activated in response to several stimuli [25]. In particular, the neutral Mg^2+^-dependent N-SMase is regulated by glutathione (GSH) [20]. Moreover, the reduction in cellular GSH levels induced by TNF-α signaling activates N-SMase [26]. Consistently, in our study, GSH was depleted in CAPE-treated C6 glioma cells (data not shown), and N-SMase was activated. However, the mechanism by which CAPE reduces GSH levels requires further investigation.

p75NTR induces cell death in primary trigeminal, sympathetic neurons as well as in Schwann and neuroblastoma cells. The signaling events that link p75NTR activation to apoptosis have begun to emerge. p75NTR-dependent apoptosis is associated with an increase in JNK activity and caspase activation [27,28]. Consistently, our results demonstrated that CAPE-induced p75NTR expression was associated with the activation of JNK and caspase 3. CAPE increased the expression of p75NTR, but not TrkA, within 6 h. The rapid increase in p75NTR might be regulated at the translational or post-translational level. However, it might also result from a decrease in p75NTR degradation, which may occur under physiological conditions. On the other hand, the NGF level also increased after treatment with CAPE. NGF exerts different effects by binding with its receptors, TrkA and p75NTR. TrkA transduces NGF signals for survival and differentiation, whereas p75NTR induces cell death. The increase in NGF and p75NTR shown in the present study indicates an amplification of apoptotic signals.

**Figure 7:**
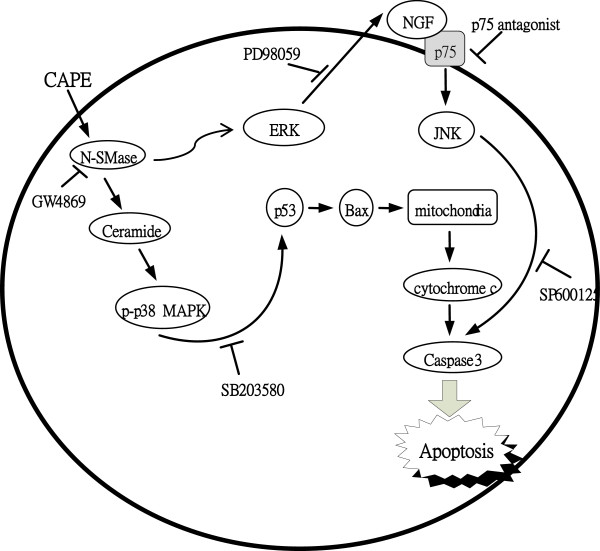
**Schematic presentation of the signaling pathways involved in CAPE-induced apoptotic pathway in C6 glioma cells.** The effect of CAPE on the MAPKs signaling triggering activation of NGF/p75NTR/JNK and p53 dependent apoptosis, which induces the N-SMase/ceramide up-regulation and increases an apoptosis cascade.

Our study indicates that the p38 MAPK/p53 activation and ERK/NGFR/NGF/JUNK pathway are two parallel pathway to activate caspase 3 and leading to cell death. As shown in Figure 3A, the MAPK signaling inhibitor (PD98059), significantly reversed the CAPE-induced expression of p75NTR and NGF at 12 h, but p38 MAPK inhibitor SB203580 and JNK inhibitor SP600125 had no effect. To determine the effect of SMase inhibitor (GW4869) also block CAPE induced MAPKs activation, we assessed whole cell lysates from CAPE-treated C6 cells by western blotting analysis (Figure 5A). GW4869 showed significantly inhibited all the CAPE-induced phosphorylation of MAPKs. Taken together, our data contribute new information about the mechanisms by which CAPE induces the apoptosis of C6 cells. We found that treatment of C6 cells with CAPE sequentially resulted in the activation of the signaling pathways mediated by N-SMase activation. Activation of the MEK/ERK pathway led to upregulation of NGF/p75NTR/JNK, whereas activation of the p38 MAPK pathway was related to an increase in p53 dependent apoptosis and increases in the activation of caspase 3 and PARP cleavage [8]. We have identified a novel role of CAPE as an effective therapeutic agent against glioma cells (Figure 7).

## Conclusion

Our previous paper demonstrated that CAPE activated p38 MAPK and that p38 kinase formed a complex with p53, resulting in the release of cytochrome C after treatment with CAPE for 0.5 h. Furthermore, we suggest that N-SMase/ceramide is the upstream target of p38 MAPK. Increasing the apoptotic potential of cancer cells through rational manipulation of ceramide levels may improve the efficacy of chemotherapy agents. Although CAPE induces apoptosis in C6 glioma cells by activating multiple signaling pathways (Figure 7), the activation of N-SMase/ceramide plays an essential role. In conclusion, the N-SMase/ceramide pathway is an important target of novel chemotherapy agents for the treatment of glioma.Our previous paper demonstrated that CAPE activated p38 MAPK and that p38 kinase formed a complex with p53, resulting in the release of cytochrome C after treatment with CAPE for 0.5 h. Furthermore, we suggest that N-SMase/ceramide is the upstream target of p38 MAPK. Increasing the apoptotic potential of cancer cells through rational manipulation of ceramide levels may improve the efficacy of chemotherapy agents. Although CAPE induces apoptosis in C6 glioma cells by activating multiple signaling pathways (Figure 7), the activation of N-SMase/ceramide plays an essential role. In conclusion, the N-SMase/ceramide pathway is an important target of novel chemotherapy agents for the treatment of glioma.

## Competing interests

The authors declare that they have no competing interests.

## Authors’ contributions

T-HT: Design, collection, assembly of data and manuscript writing, C-HS: Conception, collection, and assembly of data, W-SH: Provision of study material or patients, C-NC: Provision of study material, collection, and assembly of data, W-HL: Provision of study material, collection and assembly of data, H-CK and T-HL: Conception and design, financial support, administrative support, manuscript writing, final approval of manuscript.
